# Medicare Beneficiaries’ Perspectives on the Quality of Hospital Care and Their Implications for Value-Based Payment

**DOI:** 10.1001/jamanetworkopen.2023.19047

**Published:** 2023-06-21

**Authors:** Logan Trenaman, Mark Harrison, Jeffrey S. Hoch

**Affiliations:** 1 Centre for Health Evaluation and Outcome Sciences, St Paul’s Hospital, Vancouver, British Columbia, Canada; 2 Center for Healthcare Policy and Research, University of California, Davis, Sacramento; 3 Department of Public Health Sciences, University of California, Davis; 4 Collaboration for Outcomes Research and Evaluation, Faculty of Pharmaceutical Sciences, University of British Columbia, Vancouver, British Columbia, Canada

## Abstract

**Importance:**

Medicare’s Hospital Value-Based Purchasing (HVBP) program adjusts hospital payments according to performance on 4 equally weighted quality domains: clinical outcomes, safety, patient experience, and efficiency. The assumption that performance on each domain is equally important may not reflect the preferences of Medicare beneficiaries.

**Objective:**

To estimate the relative importance (ie, weight) of the 4 quality domains in the HVBP program from the perspective of Medicare beneficiaries and the impact of using beneficiary value weights on incentive payments for hospitals enrolled in fiscal year 2019.

**Design, Setting, and Participants:**

An online survey was conducted in March 2022. A nationally representative sample of Medicare beneficiaries was recruited through Ipsos KnowledgePanel. Value weights were estimated using a discrete choice experiment that asked respondents to choose between 2 hospitals and indicate which they preferred. Hospitals were described using 6 attributes, including (1) clinical outcomes, (2) patient experience, (3) safety, (4) Medicare spending per patient, (5) distance, and (6) out-of-pocket cost. Data analysis was performed from April to November 2022.

**Main Outcomes and Measures:**

An effects-coded mixed logit regression model was used to estimate the relative importance of quality domains. HVBP program performance was linked to Medicare payment data in the Medicare Inpatient Hospitals by Provider and Service data set and hospital characteristics from the American Hospital Association Annual Survey data set, and the estimated impact of using beneficiary value weights on hospital payments was estimated.

**Results:**

A total of 1025 Medicare beneficiaries (518 women [51%]; 879 individuals [86%] aged ≥65 years; 717 White individuals [70%]) responded to the survey. A hospital’s performance on clinical outcomes was most highly valued by beneficiaries (49%), followed by safety (22%), patient experience (21%), and efficiency (8%). Nearly twice as many hospitals would see a payment reduction when using beneficiary value weights than would see an increase (1830 vs 922 hospitals); however, the average net decrease was smaller (mean [SD], −$46 978 [$71 211]; median [IQR], −$24 628 [−$53 507 to −$9562]) than the comparable increase (mean [SD], $93 243 [$190 654]; median [IQR], $35 358 [$9906 to $97 348]). Hospitals seeing a net reduction with beneficiary value weights were more likely to be smaller, lower volume, nonteaching, and non–safety-net hospitals located in more deprived areas that served less complex patients.

**Conclusions and Relevance:**

This survey study of Medicare beneficiaries found that current HVBP program value weights do not reflect beneficiary preferences, suggesting that the use of beneficiary value weights may exacerbate disparities by rewarding larger, high-volume hospitals.

## Key Points

**Question:**

What is the relative importance (ie, weight) of the 4 quality domains in Medicare’s Hospital Value-Based Purchasing (HVBP) program from the perspective of Medicare beneficiaries and what is the estimated impact of using beneficiaries’ weights on Medicare’s incentive payments for hospitals?

**Findings:**

In this survey study of 1025 Medicare beneficiaries, clinical outcomes was the most important quality domain when choosing a hospital (weight, 49%), followed by safety (weight, 22%), patient experience (weight, 21%), and efficiency (weight, 8%).

**Meaning:**

These findings suggest that current HVBP program value weights do not reflect the preferences of Medicare beneficiaries, and using beneficiary preferences may exacerbate disparities by rewarding larger, high-volume hospitals.

## Teaser

This survey study estimates the relative importance of 4 quality domains in the Medicare Hospital Value-Based Purchasing program from the perspective of Medicare beneficiaries and quantifies the association of using beneficiary value weights with incentive payments for hospitals.

## Introduction

The US spends more on health care than any other country, and the growth in health care spending is outpacing the economy.^1,2^ This is unsustainable, leading payers to search for ways to improve the quality of care while containing costs.^3,4,5^ This includes the Centers for Medicare & Medicaid Services (CMS), the single largest payer for health care in the US.^6^ To address rising costs, the CMS has experimented with value-based payment models that hold hospitals financially accountable for the cost and the quality of care they deliver.^7,8,9^ Under value-based payment models, hospitals are financially rewarded for delivering better and more cost-effective care and are penalized for delivering poor quality or cost-ineffective care.^10^

One of the CMS’s longest running value-based payment models is the Hospital Value-Based Purchasing (HVBP) program.^11^ The HVBP operates by reducing base operating payments to hospitals by 2%, equivalent to approximately $1.9 billion in 2019, and redistributing these funds on the basis of quality and resource use measures.^12^ Hospitals can receive an incentive payment that is less than, equal to, or more than the amount withheld for that year. Redistribution of funds is based on each hospital’s total performance score, a composite measure of performance on 4 quality domains: (1) clinical care, (2) person and community engagement, (3) safety, and (4) efficiency and cost reduction. The CMS assigns equal weight to the 4 quality domains.^13^

A criticism of Medicare’s value-based payment approach is that it continues “…to measure and reward quality and safety of care in a largely provider-centric model, without having meaningfully explored the patient’s priorities in the quality framework.”^14^ This is at odds with Porter, who argued that, “Value should always be defined around the customer, and in a well-functioning health care system, the creation of value for patients should determine the rewards for all other actors in the system.”^15^ The vision of a high-value, patient-centered health care system is achievable, but will require that “patient-centered outcomes, perspectives, and preferences are explicitly incorporated into the definitions and metrics of quality, cost, and value.”^16^ When CMS assigns equal weight to the 4 quality domains in the HVBP, the assumption is that they are equally valuable. However, this assumption is not informed by evidence and may not reflect beneficiaries’ perspectives on what constitutes high-value care. One way of aligning the incentives in the HVBP with value from the patient’s perspective is to have Medicare beneficiaries define the relative importance of each domain (ie, the domain weights).

Our study addresses 2 questions. First, what is the relative importance of the 4 quality domains in the HVBP from the perspective of Medicare beneficiaries (referred to hereafter as *beneficiary value weights*)? Second, what is the estimated impact of using beneficiary value weights on incentive payments for hospitals enrolled in the HVBP in fiscal year 2019?

## Methods

We used an online survey that included a discrete choice experiment (DCE) to estimate beneficiary value weights.^17^ The DCE method has a strong theoretical underpinning^18,19^ and is designed to elicit the relative value of characteristics (or attributes) of a good or service rather than the good or service as a whole.^20^ More than 600 DCEs quantifying preferences for aspects of health or health care have been published in the literature.^21^ This survey study was approved by the University of California, Davis, institutional review board, and all participants provided electronic informed consent. The survey was developed following recommendations from the International Society for Pharmacoeconomics and Outcomes Research (ISPOR) reporting guideline and reported following best practices from the American Association for Public Opinion Research (AAPOR) reporting guideline.^17,22^

### Study Population and Data Collection

Medicare beneficiaries were recruited through Ipsos KnowledgePanel*,* a US-based online panel that includes approximately 60 000 members. Members are recruited through address-based probability sampling based on the delivery sequence file of the US postal services, which covers nearly 99% of the US population. Participation is encouraged through an incentive program that includes special raffles and sweepstakes with cash rewards and other prizes.

The survey was fielded between March 15 and 29, 2022. The target population included preidentified panelists who reconfirmed that they were currently enrolled in Medicare. The sampling strategy focused on identifying a representative sample based on age (<65 years vs ≥65 years), gender (male or female), and race and ethnicity (American Indian or Alaska Native, Asian or Pacific Islander, Black or African American, Hispanic, non-Hispanic White, and any other race), with selective oversampling of 3 racial and ethnic groups (Black or African American, Asian or Pacific Islander, and Hispanic). Race and ethnicity are included in the study because they have been shown to be associated with selecting hospitals and physicians. Quotas were estimated on the basis of Medicare enrollment statistics for 2019. Respondents’ sociodemographic characteristics were patient-reported and obtained directly from Ipsos. These included insurance provider, age, education, race and ethnicity, gender, household size, housing type and ownership status, household income, marital status, metropolitan area status, state of residence, and current employment status.

### Survey Development

**Figure 1.**
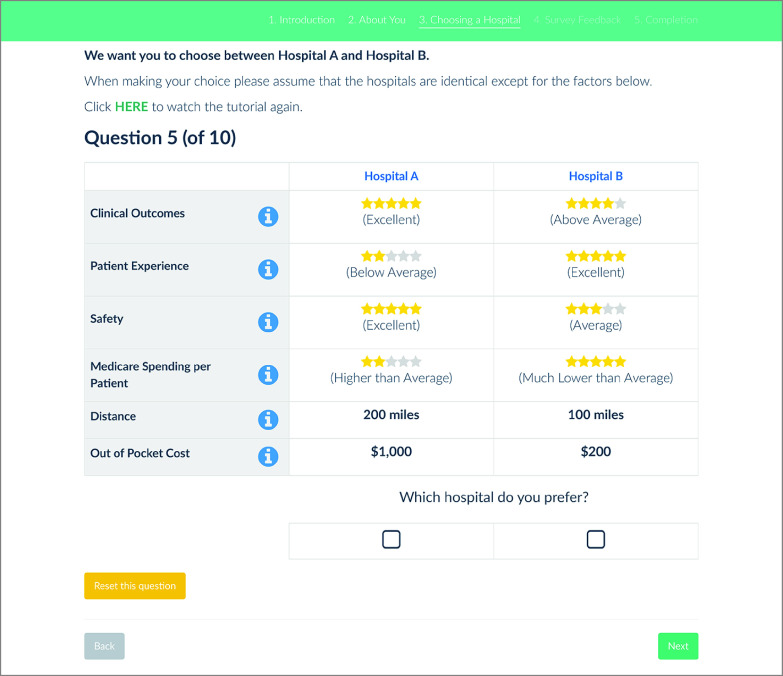
Example of a Discrete Choice Experiment Choice Set

The DCE presented beneficiaries with a choice between 2 hypothetical hospitals, described using the domains of quality used in the HVBP. Respondents were asked to imagine that they needed to choose a new hospital to seek care and to indicate which hospital they prefer (Figure 1). Preferences were assessed over the full range of potential attributes and levels (Table 1). Four of the 6 attributes were derived from the domains of the HVBP, which included clinical outcomes, patient experience (person and community engagement), safety, and Medicare spending per patient (efficiency and cost reduction). The domain attribute descriptions were derived from Medicare’s patient-facing websites, whereas levels were represented by Medicare star ratings, which ranged from 1 to 5 stars, with more stars indicating better ratings (eTable 1 in Supplement 1). The remaining 2 attributes were distance and out-of-pocket cost, with levels derived from a previous DCE that used star ratings.^23^ The survey was refined through focus groups, in-depth think-aloud interviews with Medicare beneficiaries, and a pilot study of Medicare beneficiaries recruited through Amazon’s Mechanical Turk. After the DCE, the survey asked several debriefing questions related to respondents’ understanding and difficulty of the choice task, whether there were any attributes they ignored (known as attribute nonattendance), and a task that ranked attributes by importance (see the eFigure in Supplement 1).

**Table 1.** Respondent Characteristics

Characteristic	Respondents, No. (%) (N = 1025)
Age group, y	
18-34	8 (1)
35-44	22 (2)
45-54	37 (4)
55-64	79 (8)
65-74	565 (55)
≥75	314 (31)
Gender	
Female	518 (51)
Male	507 (49)
Race	
American Indian or Alaska Native	11 (1)
Asian or Pacific Islander	63 (6)
Black or African American	211 (21)
White	717 (70)
≥2 Races	23 (2)
Ethnicity	
Black, Non-Hispanic	206 (20)
Hispanic	204 (20)
White, Non-Hispanic	535 (52)
≥2 Races, Non-Hispanic	14 (1)
Other, non-Hispanic^a^	66 (6)
Highest educational attainment	
No high school diploma or GED	71 (7)
High school graduate (high school diploma or the equivalent GED)	274 (27)
Some college or associate’s degree	328 (32)
Bachelor’s degree	163 (16)
Master’s degree or higher	189 (18)
Annual household income, $	
<10 000	23 (2)
10 000 to 24 999	145 (14)
25 000 to 49 999	246 (24)
50 000 to 74 999	194 (19)
75 000 to 99 999	135 (13)
100 000 to 149 999	147 (14)
≥150 000	135 (13)
Marital status	
Divorced	143 (14)
Never married	118 (12)
Now married	618 (60)
Separated	19 (2)
Widowed	127 (12)
Metropolitan area status	
Metropolitan	901 (88)
Nonmetropolitan	124 (12)
Region	
Midwest	188 (18)
Northeast	160 (16)
South	439 (43)
West	238 (23)
Employment status	
Not working	869 (85)
Working full time	49 (5)
Working part time	107 (10)
Health insurance status	
Medicare, for people aged ≥65 y, or people with certain disabilities	1025 (100)
Medicaid, Medical Assistance, or another government-assistance plan	93 (9)
Insurance through a current or former employer or union	179 (17)
Insurance purchased directly from an insurance company	141 (14)
TRICARE or other military health care	50 (5)
VA (enrolled for VA health care)	47 (5)
Indian Health Service or any other type of health insurance or health coverage plan	48 (5)

Abbreviations: GED, general educational development; VA, Veterans Affairs.

^a^ Other includes any other race not specified.

### Experimental Design

It is often impractical to include all possible combinations of attributes and levels in a DCE given the number of potential combinations. The dcreate algorithm in Stata statistical software version 15.1 (StataCorp) was used to select a D-efficient fractional factorial experimental design using bayesian priors from the pilot survey.^24^ The final design included 45 questions that were blocked into 5 versions comprising 9 questions. Participants were randomly allocated to 1 version. The DCE included 2 practice questions (at the start) and a consistency check (duplicate question at the end) that were not scored.

### Hospital Performance and Medicare Payment Data

We used 2 publicly available data sources to estimate the impact of beneficiary value weights on hospital reimbursement. Unweighted domain scores for hospitals enrolled in the HVBP in fiscal year 2019 were obtained from the HVBP TPS table from the CMS Hospital Compare data set.^25^ There were 2786 hospitals that participated in the HVBP program in 2019. Medicare expenditures by hospital for fiscal year 2019 were obtained from the Medicare Inpatient Hospitals by Provider and Service data set from the CMS.^26^ This data set contains information on services provided to fee-for-service Medicare beneficiaries through Medicare Part A (Hospital Insurance) at more than 3000 hospitals that received payments through Medicare’s Inpatient Prospective Payment System (IPPS). It provides hospital-specific estimates of the number and average cost to Medicare for discharges by Medicare Severity Diagnosis Related Group. In total, this data set includes more than 7 million discharges representing 75% of all Medicare discharges paid through the IPPS.

Data on the characteristics of Medicare hospitals were derived from the 2019 American Hospital Association (AHA) Annual Survey and the 2019 fiscal year CMS Impact datafile. The AHA datafile provided information on ownership status, bed size, admission volume, accreditation, membership in the Council of Teaching Hospitals, provision of transplant services, level I trauma center status, inpatient surgical procedures per bed, and nurse-to-bed and resident-to-bed ratios. We used 5-digit county Federal Information Processing System codes from the AHA annual survey to calculate each hospital’s Area Deprivation Index using the sociome R package.^27^ The CMS Impact datafile provided information on each hospital’s core based statistical area, case mix index (CMI), and percentage of disproportionate share patients. CMI indicates the average complexity of patients treated by the hospital, with a higher CMI indicating more complex admissions and potentially more ill patients.^28^ The disproportionate shares percentage is a measure of whether the hospital serves a disproportionate number of low-income patients and receives payments from the CMS to cover uninsured patients.^29^ Those in the highest quartile are considered safety-net hospitals.^30^

### Statistical Analysis

Data analysis was performed from April to November 2022. The primary analysis used an effects-coded mixed logit regression model to estimate preferences. For all analyses, we weighted respondents to be representative of the Medicare population. The design weights for respondents who reconfirmed as having Medicare health insurance coverage were raked to match the following geodemographic distributions of the US population aged 18 years and older with annual Medicare health insurance coverage: race and ethnicity by age group, gender by age group, US Census region, metropolitan status, and household income. The needed benchmarks were obtained from the March 2021 Current Population Survey Annual Social and Economic Supplement (eTable 2 in Supplement 1). We estimated beneficiary value weights by comparing the range of coefficients for each of the 4 quality domain attributes (from least to most preferred level) as a share of the total range across all these 4 attributes.^31^

We linked hospital performance, payment, and characteristic data using each hospital’s unique Medicare identification number. For each participating hospital, we estimated total base operating payments through the IPPS, the total reduction in base operating payments (2%), and the total incentive payments using equal weights and beneficiary value weights.^12^ These calculations were based on publicly available information from the CMS.^32^ We conducted 2 sensitivity analyses in subsamples that (1) passed the consistency check and (2) self-reported that they understood the concept of making choices between the different hospitals.

## Results

### Survey Respondents

A total of 1835 eligible Medicare beneficiaries were invited to participate, and 1025 (518 women [51%]; 879 respondents aged ≥65 years [86%]; 717 White individuals [70%]) completed the survey (Table 1), for a response rate of 56%. The sample included beneficiaries from 50 states and the District of Columbia. The distributions of gender, age, income, education, and geographic location were broadly representative of the Medicare beneficiary population. As expected, we had a higher proportion of beneficiaries who identified as Black or African American, Asian, and Hispanic because of our sampling strategy. The impact of using sampling weights on the sample characteristics is presented in eTable 3 in Supplement 1.

### Relative Importance of Quality Domains From the Perspective of Medicare Beneficiaries

**Figure 2.**
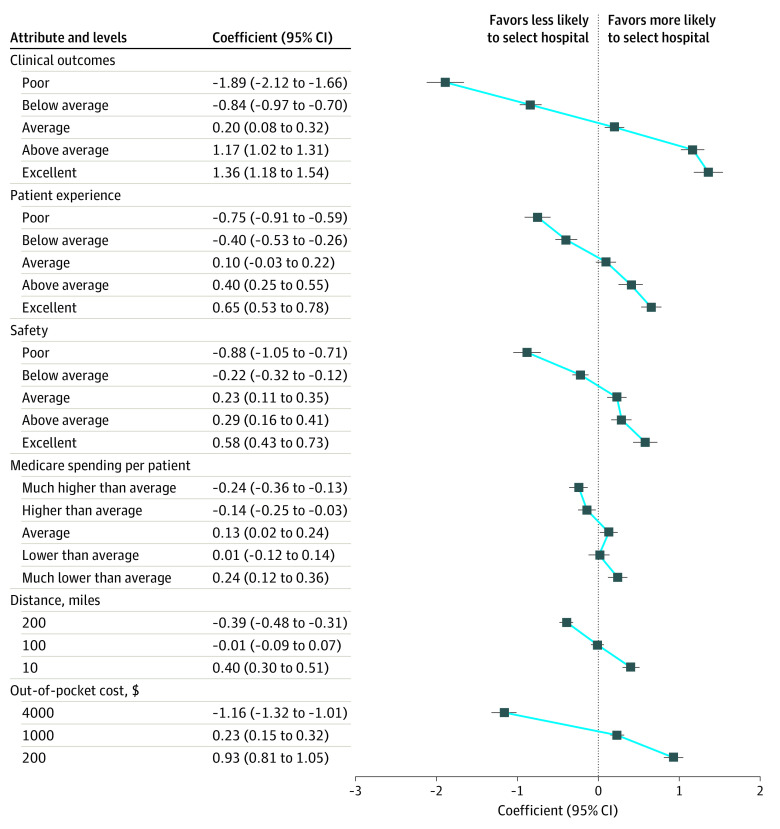
Estimated Impact of Medicare’s Hospital Value-Based Purchasing Quality Domains, Distance, and Cost on Hospital Selection

Attribute and level coefficients for the 4 HVBP domains, distance, and cost are presented in Figure 2, and the full model results are reported in eTable 4 in Supplement 1. A coefficient greater than 0 indicates that including the attribute level in a hospital profile increases the likelihood that a hospital would be selected. For example, the coefficient for a 5-star rating on clinical outcomes was 1.36. This means that a hospital with a 5-star rating on clinical outcomes was more likely to be chosen if it included that attribute level. A coefficient less than 0 indicates including that attribute level in a hospital profile decreases the likelihood that a hospital would be selected. For example, the coefficient for a 1-star rating on clinical outcomes was −1.89. The means that a hospital with a 1-star rating on clinical outcomes was less likely to be chosen if it included that attribute level. Broadly speaking, a star rating of average or above (3 stars or above) made a profile more likely to be chosen when a rating below average (2 stars or lower) made a profile less likely to be chosen. Beneficiary value weights for the domains were derived from the difference in coefficients between and 1-star and 5-star rating. Clinical outcomes received the highest weight (49%; coefficient range, −1.89 to 1.36), followed by safety (22%; coefficient range, −0.88 to 0.58), patient experience (21%; coefficient range, −0.75 to 0.65), and Medicare spending per patient (8%; coefficient range, −0.24 to 0.24).

With respect to the sensitivity analysis, a total of 875 respondents (85.4%) passed the consistency check, whereas 901 (87.9%) reported understanding the choice task. The results of sensitivity analyses based on these 2 subsamples are presented in eTable 5 and eTable 6 in Supplement 1. These analyses demonstrated that our estimated beneficiary value weights are robust (data not shown).

### Estimated Impact of Beneficiary Value Weights on Hospital Reimbursement

**Figure 3.**
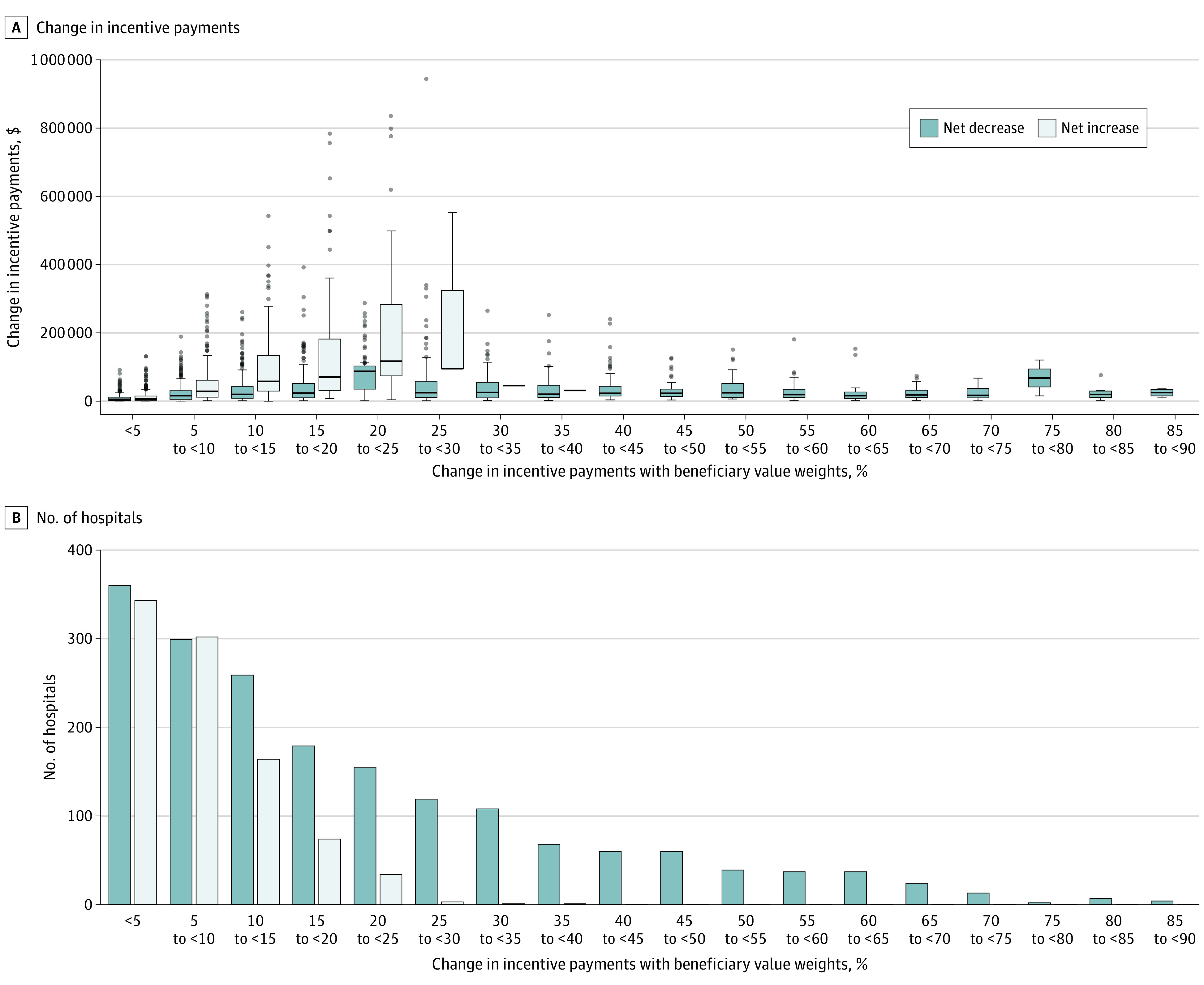
Estimated Impact of Using Beneficiary Preferences on Incentive Payments and Number of Hospitals Affected Box plots (A) summarize the distribution of the data. This includes the first quartile or 25th percentile (lower end of box), median (horizontal line in the box), and third quartile or 75th percentile (upper end of box). The boundaries of the whiskers are based on 1.5 times the IQR (ie, the distance between the 25th and 75th percentiles. The upper whisker is drawn at the largest observed data point within 1.5 times the IQR above the 75th percentile, whereas the lower whisker is drawn at minimum observed data point that is within 1.5 times the IQR below the 25th percentile. The plot also includes outliers that fall beyond these whiskers (points). Three hospitals in the 20% to less than 25% category are beyond the range of this graph and would see net increases of $1.72 million, $1.79 million, and $2.80 million, respectively. In both panels, the change in incentive payments were calculated as the difference in incentive payments between beneficiary and current weights divided by the total amount withheld by Medicare for incentive payments (2% of total Medicare payments).

We linked performance and payment data for 2752 hospitals and more than $80 billion in payments administered through the IPPS. Using beneficiary value weights would result in a reallocation of nearly $86 million. Nearly double the number of hospitals would see a payment reduction when using beneficiary value weights than would see an increase (1830 vs 922 hospitals); however, the net decrease in incentive was smaller (mean [SD], −$46 978 [$71 211]; median [IQR], −$24 628 [−$53 507 to −$9562]) than the comparable increase (mean [SD], $93 243 [$190 654]; median [IQR], $35 358 [$9906 to $97 348]). This was primarily associated with large absolute increases in incentive payments (Figure 3A) in relatively few high-volume hospitals (Figure 3B). Of the 2752 hospitals with performance and payment data, 35 (1.3%) were not included in the 2019 AHA database and 6 (0.2%) were not included in the 2019 CMS Impact file. Table 2 reports the characteristics of hospitals that would see a net increase and net decrease in incentive payments when using beneficiary value weights. Overall, those seeing a net reduction were more likely to be in less populated areas and have fewer hospital beds and admissions and serve less-complex patients, as measured by CMI. Furthermore, they were less likely to be a safety-net hospital, teaching hospital, have accreditation through the Joint Commission or American College of Surgeons for Cancer, or be a member of the Council of Teaching Hospitals (Table 2).

**Table 2.** Hospital Characteristics by Incentive Change When Using Beneficiary Value Weights

Characteristic	Hospitals, No. (%)	
	Net decrease	Net increase
No. of hospitals	1830 (66.5)	922 (33.5)
Ownership^a^		
Government, nonfederal	251 (13.9)	94 (10.3)
Nongovernment, not-for-profit	1240 (68.7)	604 (66.2)
Investor-owned (for profit)	307 (17.0)	214 (23.5)
Government, federal	7 (0.4)	0 (0.0)
Core-based statistical area^b^		
Metropolitan (>50 000 people)	1234 (68.4)	853 (93.5)
Micropolitan (10 000-50 000 people)	424 (23.5)	49 (5.4)
Rural (<10 000 people)	147 (8.1)	10 (1.1)
Hospital bed size^a^		
Small (<100)	662 (36.7)	76 (8.3)
Medium (100-399)	983 (54.5)	558 (61.2)
Large (>400)	160 (8.9)	278 (30.5)
Total hospital admissions^a^		
Quartile 1 (≤3423)	619 (34.3)	61 (6.7)
Quartile 2 (3424-7778)	509 (28.2)	170 (18.6)
Quartile 3 (7779-15 325)	399 (22.1)	280 (30.7)
Quartile 4 (>15 325)	278 (15.4)	401 (44.0)
Joint Commission Accreditation^a^	1416 (78.4)	798 (87.5)
American College of Surgeons Cancer Accreditation^a^	669 (37.1)	494 (54.2)
Member of the Council of Teaching Hospitals^a^	59 (3.3)	170 (18.6)
Transplant services^a^	135 (7.4)	111 (12.0)
Level I trauma center^a^	66 (3.6)	157 (17.0)
Resident-to-bed ratio^a^		
Nonteaching (0.000)	1282 (70.2)	423 (45.9)
Very minor teaching (0.001-0.049)	188 (10.3)	106 (11.5)
Minor teaching (0.050-0.249)	250 (13.7)	202 (21.9)
Major teaching (0.250-0.599)	72 (3.9)	138 (15.0)
Very major teaching (>0.600)	33 (1.8)	52 (5.6)
Nurse-to-bed ratio^a^		
Quartile 1 (≤1.4620)	481 (26.6)	199 (21.8)
Quartile 2 (1.4621-1.9020)	447 (24.8)	232 (25.4)
Quartile 3 (1.9021-2.4333)	422 (23.4)	257 (28.2)
Quartile 4 (>2.4333)	455 (25.2)	224 (24.6)
Inpatient surgical procedures per bed^a^		
Quartile 1 (≤7.570)	503 (27.9)	177 (19.4)
Quartile 2 (7.571-10.774)	461 (25.5)	218 (23.9)
Quartile 3 (10.775-14.612)	415 (23.0)	264 (28.9)
Quartile 4 (>14.612)	426 (23.6)	253 (27.7)
Case mix index^b^		
Quartile 1 (≤1.3897)	460 (25.2)	74 (8.0)
Quartile 2 (1.3898-1.5816)	554 (30.4)	201 (21.8)
Quartile 3 (1.5817-1.8000)	474 (26.0)	319 (34.6)
Quartile 4 (>1.8000)	337 (18.5)	327 (35.5)
Disproportionate shares hospital percentage^b^		
Quartile 1 (≤0.19090)	405 (22.2)	160 (17.4)
Quartile 2 (0.19091-0.27910)	513 (28.1)	212 (23.0)
Quartile 3 (0.27911-0.37710)	492 (27.0)	252 (27.4)
Quartile 4 (>0.37710)^c^	415 (22.7)	297 (32.2)
Area deprivation index, mean (SD)^a^	96.2 (20.8)	92.2 (19.1)

^a^ Data are included for 2717 hospitals. Variable was missing from the 2019 AHA Annual Survey for 35 hospitals (1.3%).

^b^ Data are included for 2746 hospitals. Variable was missing from the 2019 CMS Impact File for 6 hospitals (0.2%).

^c^ Serves as an indicator of safety-net hospital.

## Discussion

This survey study used a rigorous preference elicitation method and sampling approach to estimate weights for the 4 quality domains used in the HVBP from the perspective of Medicare beneficiaries. Our results suggest that beneficiaries do not value all domains equally and, instead, place greater weight on clinical outcomes, primarily at the expense of efficiency. We estimated that using beneficiary value weights in the HVBP in 2019 would have resulted in nearly $86 million in incentive payments to hospitals being reallocated. Importantly, our analysis suggests that using beneficiary value weights would penalize smaller rural hospitals.

The CMS uses quality weights to aggregate hospital performance for value-based payment programs and to generate overall star ratings. Schang et al note that, “arriving at explicit trade-offs between different healthcare quality measures—and thus exact specifications of weights—is highly contentious.”^33^ In many cases, the HVBP program included, equal weights are chosen without justification.^34^ However, decisions about how measures and domains are aggregated into composite scores can substantially affect assessments of hospital performance.^35,36^ Here, we have generated empirical evidence on beneficiary preferences according to how they trade off between the quality domains.

An important consideration is whether hospitals respond to the incentives to improve the quality of care. A 2020 systematic review^37^ found limited evidence that Medicare’s HVBP program has improved quality or outcomes. One explanation offered for this finding was that the incentives in the HVBP program are small, which may not be sufficient to result in changes in care.^38^ If the current incentives are insufficient to motivate hospitals to make investments to improve care, it is unlikely that using beneficiary value weights within the current incentive structure (ie, 2% of payments) would motivate change. Indeed, the change in incentive payments when modifying value weights for most hospitals actually represents a relatively modest amount of the 2% that is withheld. Regardless, we have highlighted which aspects of quality are most important to Medicare beneficiaries and can inform value-based payment reforms and quality improvement activities.

Evidence suggests that patient perspectives on value often differ from those of payers and hospitals.^39,40^ It is understandable that beneficiaries place greater weight on outcomes, experience, and safety at the expense of efficiency given that the cost is borne by the CMS. By contrast, the CMS has an obligation to ensure that resources are allocated efficiently so that it can improve the outcomes, safety, and experience of care for as many beneficiaries as possible. Integrating these perspectives is possible. For example, beneficiary value weights could be used for the outcomes, safety, and patient experience domains, whereas Medicare could set the weight for the efficiency domain. Furthermore, the perspectives of other key stakeholders, such as hospitals, could be considered and incorporated into the program design.

Value weights represent one way in which beneficiary perspectives can be incorporated into the design of value-based payment programs. Beneficiary perspectives could also inform decisions about the quality domains and selection of measures used to evaluate them. Beneficiary perspectives can also be through the use of patient-reported outcomes and patient-reported experience measures.^41^ The Hospital Consumer Assessment of Healthcare Providers and Systems patient-reported experience measure is already used the HVBP to assess the patient experience domain. This could be expanded to include routinely collected patient-reported outcomes to evaluate the outcomes or safety domains.

A long-standing concern with the HVBP program is the potential for it to establish or exacerbate disparities in care.^42,43^ For example, hospitals with a higher proportion of Black patients are more likely to be penalized under the HVBP, even when controlling for hospital characteristics and safety-net status.^44^ Our analysis suggests that using beneficiary value weights might exacerbate disparities in some respects (eg, penalizing smaller, rural, low volume, nonteaching hospitals that serve more deprived areas), and reduce disparities in others (eg, rewarding larger hospitals who are more likely to be safety-net institutions). Commentators suggest that a major reason that the CMS’s value-based payment programs have failed to improve health equity is because it was not considered in the design and implementation.^45^ Establishing health equity as an explicit objective of value-based payment reforms and tying equity to payments can be accomplished by incorporating equity measures into assessment and/or modifying the payment criteria to assign a greater weight to quality improvement rather than quality achievement.^42,46^ Notably, the CMS is taking steps to incorporate equity in its value-based payment programs, having recently announced the Accountable Care Organization Realizing Equity, Access, and Community Health model, which explicitly aims to improve both equity and value.^45^

### Strengths and Limitations

There are several strengths of this analysis. The relative importance of quality domains was evaluated through a survey in a large, nationally representative sample of Medicare beneficiaries. We preidentified a sample of Medicare beneficiaries from an internet panel and reaffirmed their status before asking them to participate. Our sampling approach used address-based methods from the US postal service delivery file, which covers all US households, regardless of whether they have a telephone. Furthermore, panelists must be invited (rather than allowed to opt-in) and only complete a few surveys per month to avoid fatigue.

Our study also has several potential limitations. First, the survey was conducted in English only. This may have biased results, because English language learners may have been less likely to respond. Second, our analysis used publicly available data on IPPS payments. This database captures approximately 75% of all IPPS payments but redacts diagnoses with 10 or fewer discharges by hospital. Consequently, we may have underestimated the impact of beneficiary value weights on incentive payments, and this would disproportionately affect low-volume hospitals.

## Conclusions

In this study, the CMS’s current approach of equally weighting the 4 quality domains in the HVBP program did not reflect the preferences of Medicare beneficiaries. Our findings suggest that using beneficiary value weights has important implications from an equity perspective and would disadvantage smaller, lower volume, nonteaching, non–safety-net hospitals located in more deprived areas that serve less-complex patients (eg, rural hospitals).

## Notes

Supplement 1. **eTable 1.** Description of Attribute and Levels **eFigure.** Survey Screenshots **eTable 2.** Geodemographic Benchmarks for Generating Weights for Medicare Population **eTable 3.** Unweighted and Weighted Respondent Characteristics **eTable 4.** Results From Effects-Coded Mixed Logit Model **eTable 5.** Results From Effects-Coded Mixed Logit Model (‘Consistent’ Subsample) **eTable 6.** Results From Effects-Coded Mixed Logit Model (‘Understood’ Subsample)

Supplement 2.
Data Sharing Statement
