# Supplementary material for: Medicare Beneficiaries’ Perspectives on the Quality of Hospital Care and Their Implications for Value-Based Payment
Source: JAMA Netw Open. 2023 Jun 21;6(6):e2319047. doi: 10.1001/jamanetworkopen.2023.19047 (PMC10285577; doi:10.1001/jamanetworkopen.2023.19047)
Supplement: Supplement 1. — eTable 1. Description of Attribute and Levels eFigure. Survey Screenshots eTable 2. Geodemographic Benchmarks for Generating Weights for Medicare Population eTable 3. Unweighted and Weighted Respondent Characteristics eTable 4. Results From Effects-Coded Mixed Logit Model eTable 5. Results From Effects-Coded Mixed Logit Model (‘Consistent’ Subsample) eTable 6. Results From Effects-Coded Mixed Logit Model (‘Understood’ Subsample) [file jamanetwopen-e2319047-s001.pdf]

Supplemental Online Content

Trenaman L, Harrison M, Hoch JS. Medicare beneficiaries' perspectives on the quality of hospital care and their implications for value-based payment. *JAMA Netw Open*. 2023;6(6):e2319047. doi:10.1001/jamanetworkopen.2023.19047

eTable 1. Description of Attribute and Levels

eFigure. Survey Screenshots

eTable 2. Geodemographic Benchmarks for Generating Weights for Medicare Population

eTable 3. Unweighted and Weighted Respondent Characteristics

eTable 4. Results From Effects-Coded Mixed Logit Model

eTable 5. Results From Effects-Coded Mixed Logit Model ('Consistent' Subsample)

eTable 6. Results From Effects-Coded Mixed Logit Model ('Understood' Subsample)

This supplemental material has been provided by the authors to give readers additional information about their work.

© 2023 Trenaman L et al. *JAMA Network Open.*

eTable 1. Description of Attribute and Levels

Attribute	Description	Levels
Clinical Outcomes	Good clinical outcomes are more likely when patients receive high-quality care in hospital and when they transition to an outpatient setting. For example, patients in a hospital with better clinical outcomes may be less likely to die in the 30 days after entering the hospital for a specific condition (like pneumonia or heart attack) or less likely to have a complication from a specific procedure (like infection following hip replacement).	☆ (Poor)<br>☆ ☆ (Below Average)<br>☆ ☆ ☆ (Average)<br>☆ ☆ ☆ ☆ (Above Average)<br>☆ ☆ ☆ ☆ ☆ (Excellent)
Patient Experience	This factor measures the experiences of adult patients who had a recent hospital stay. The patient experience score captures things like the communication and responsiveness of hospital staff and the cleanliness and quietness of the hospital environment.	☆ (Poor)<br>☆ ☆ (Below Average)<br>☆ ☆ ☆ (Average)<br>☆ ☆ ☆ ☆ (Above Average)<br>☆ ☆ ☆ ☆ ☆ (Excellent)
Safety	This factor measures things that impact patient well-being and outcomes while in hospital. Patients who received high-quality care in hospital will likely have improved outcomes. For example, patients in a hospital with better safety outcomes may be at lower risk of in-hospital falls, developing bed sores, or developing an infection.	☆ (Poor)<br>☆ ☆ (Below Average)<br>☆ ☆ ☆ (Average)<br>☆ ☆ ☆ ☆ (Above Average)<br>☆ ☆ ☆ ☆ ☆ (Excellent)
Medicare Spending per Patient	This factor measures how much Medicare spends per patient at this hospital compared to all hospitals across the country. Less spending can be a good thing. Hospitals that spend less get better ratings because they can provide care for more patients.	☆ (Much Higher than Average)<br>☆ ☆ (Higher than Average)<br>☆ ☆ ☆ (Average)<br>☆ ☆ ☆ ☆ (Lower than Average)<br>☆ ☆ ☆ ☆ ☆ (Much Lower than Average)
Distance	This factor describes the distance from your home to the hospital.	10 miles<br>100 miles<br>200 miles
Out of Pocket Cost	This factor describes your total out of pocket costs at this hospital. Assume that this is the amount that you will be responsible for paying, that would not be covered by your insurance.	$200<br>$1000<br>$4000

© 2023 Trenaman L et al. *JAMA Network Open.*

eFigure 1. Survey Screenshots

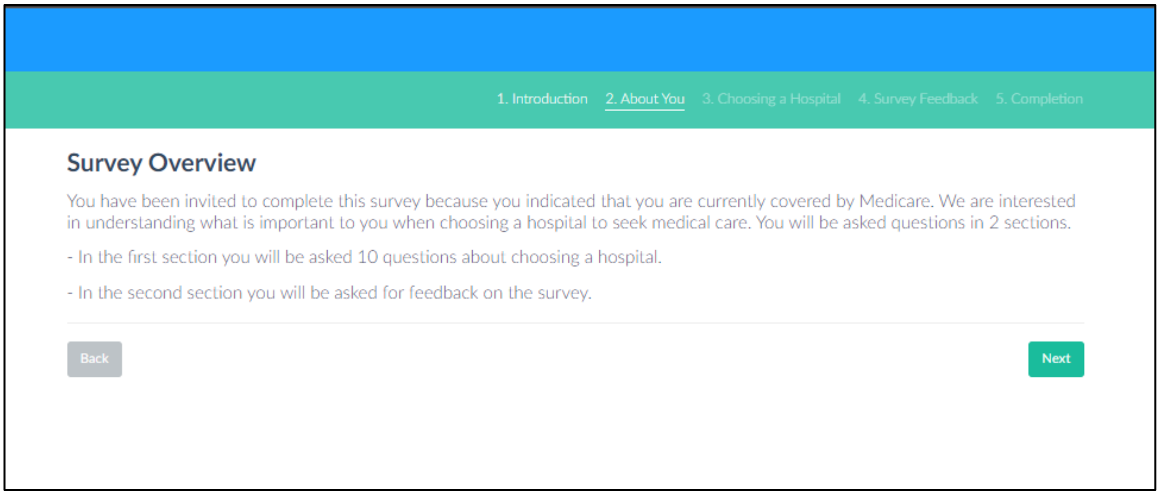

1. Introduction 2. About You 3. Choosing a Hospital 4. Survey Feedback 5. Completion

Survey Overview

You have been invited to complete this survey because you indicated that you are currently covered by Medicare. We are interested in understanding what is important to you when choosing a hospital to seek medical care. You will be asked questions in 2 sections.

- In the first section you will be asked 10 questions about choosing a hospital.

- In the second section you will be asked for feedback on the survey.

Back Next

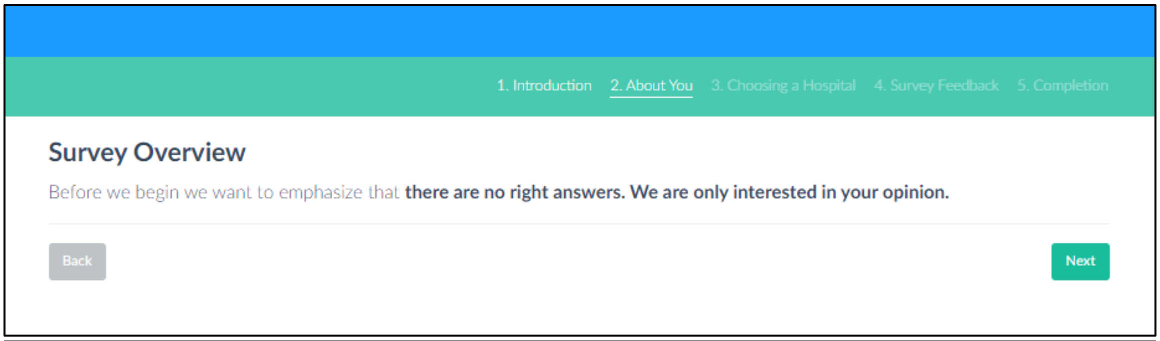

1. Introduction 2. About You 3. Choosing a Hospital 4. Survey Feedback 5. Completion

Survey Overview

Before we begin we want to emphasize that **there are no right answers. We are only interested in your opinion.**

Back Next

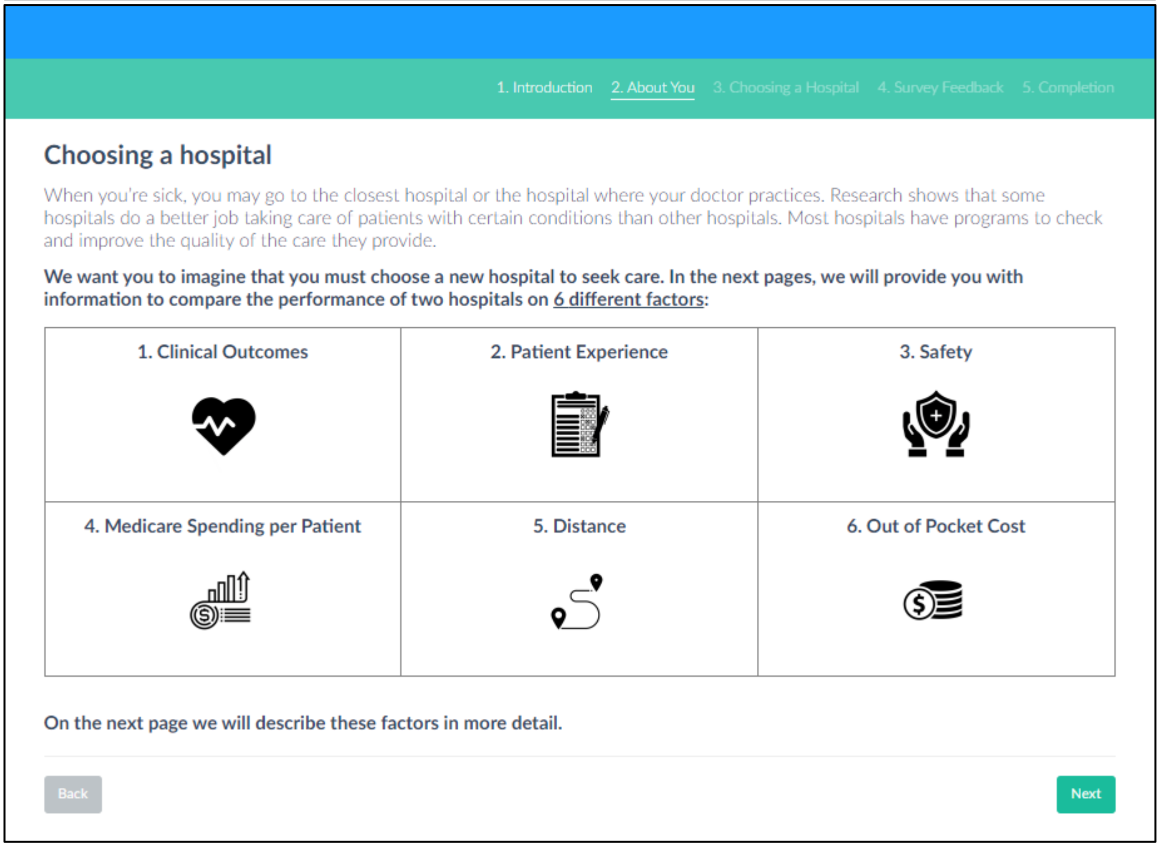

1. Introduction 2. About You 3. Choosing a Hospital 4. Survey Feedback 5. Completion

Choosing a hospital

When you're sick, you may go to the closest hospital or the hospital where your doctor practices. Research shows that some hospitals do a better job taking care of patients with certain conditions than other hospitals. Most hospitals have programs to check and improve the quality of the care they provide.

We want you to imagine that you must choose a new hospital to seek care. In the next pages, we will provide you with information to compare the performance of two hospitals on 6 different factors:

1. Clinical Outcomes	2. Patient Experience	3. Safety
4. Medicare Spending per Patient	5. Distance	6. Out of Pocket Cost

On the next page we will describe these factors in more detail.

Back Next

© 2023 Trenaman L et al. *JAMA Network Open.*

1. Introduction 2. About You 3. Choosing a Hospital 4. Survey Feedback 5. Completion

Choosing a hospital

The table below describes the six factors in more detail.

	**1. Clinical Outcomes**<br>Good clinical outcomes are more likely when patients receive high quality care in hospital and when they transition to an outpatient setting. For example, patients in a hospital with better clinical outcomes may be less likely to die in the 30 days after entering the hospital for a specific condition (like pneumonia or heart attack) or less likely to have a complication from a specific procedure (like infection following hip replacement).<br>Hospitals' performance will be rated from 1 to 5 stars.
	**2. Patient Experience**<br>This factor measures the experiences of adult patients who had a recent hospital stay. The patient experience score captures things like the communication and responsiveness of hospital staff and the cleanliness and quietness of the hospital environment.<br>Hospitals' performance will be rated from 1 to 5 stars.
	**3. Safety**<br>This factor measures things that impact patient well-being and outcomes while in hospital. Patients who received high-quality care in hospital will likely have improved outcomes. For example, patients in a hospital with better safety outcomes may be at lower risk of in-hospital falls, developing bed sores, or developing an infection.<br>Hospitals' performance will be rated from 1 to 5 stars.
	**4. Medicare Spending per Patient**<br>This factor measures how much Medicare spends per patient at this hospital compared to all hospitals across the country. Less spending can be a good thing. **Hospitals that spend less get better ratings because they can provide care for more patients.**<br>Hospitals' performance will be rated from 1 to 5 stars.
	**5. Distance**<br>This factor describes the distance from your home to the hospital.<br>Hospitals will either be **10 miles**, **100 miles**, or **200 miles** from your home.
	**6. Out of Pocket Cost**<br>This factor describes your total out of pocket costs at this hospital. Assume that this is the amount that you will be responsible for paying, that would not be covered by your insurance.<br>Hospitals will either cost you **$200**, **$1,000**, or **$4,000**.

Back Next

© 2023 Trenaman L et al. *JAMA Network Open.*

1. Introduction 2. About You 3. Choosing a Hospital 4. Survey Feedback 5. Completion

We want you to choose between Hospital A and Hospital B.

When making your choice please assume that the hospitals are identical except for the factors below.

Click **HERE** to watch the tutorial again.

Question 1 (of 10)

	Hospital A	Hospital B
Clinical Outcomes	(Below Average)	(Poor)
Patient Experience	(Excellent)	(Above Average)
Safety	(Poor)	(Average)
Medicare Spending per Patient	(Average)	(Much Lower than Average)
Distance	10 miles	200 miles
Out of Pocket Cost	$4,000	$1,000

Which hospital do you prefer?

Reset this question

Back Next

1. Introduction 2. About You 3. Choosing a Hospital 4. Survey Feedback 5. Completion

In this section you will be asked to give us some feedback on the previous questions.

Back Next

© 2023 Trenaman L et al. *JAMA Network Open.*

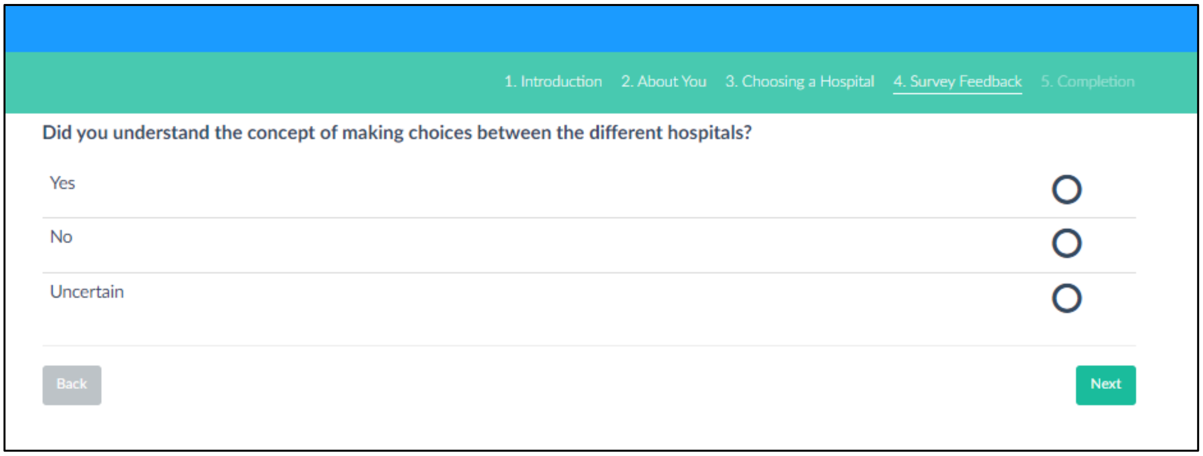

1. Introduction 2. About You 3. Choosing a Hospital 4. Survey Feedback 5. Completion

Did you understand the concept of making choices between the different hospitals?

- Yes
- No
- Uncertain

Back Next

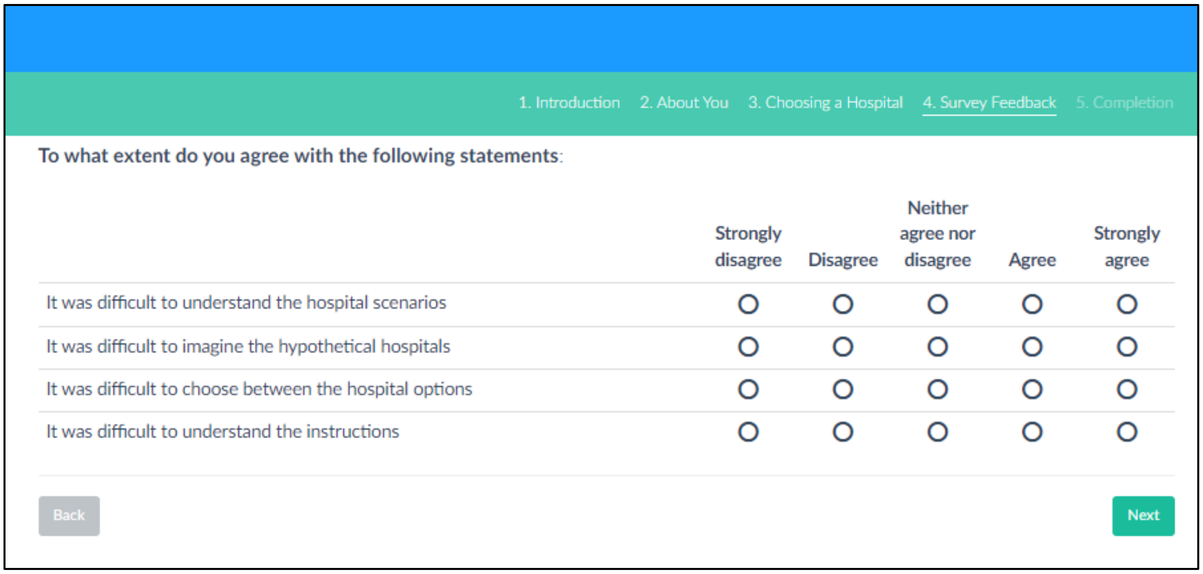

1. Introduction 2. About You 3. Choosing a Hospital 4. Survey Feedback 5. Completion

To what extent do you agree with the following statements:

	Strongly disagree	Disagree	Neither agree nor disagree	Agree	Strongly agree
It was difficult to understand the hospital scenarios	○	○	○	○	○
It was difficult to imagine the hypothetical hospitals	○	○	○	○	○
It was difficult to choose between the hospital options	○	○	○	○	○
It was difficult to understand the instructions	○	○	○	○	○

Back Next

1. Introduction 2. About You 3. Choosing a Hospital 4. Survey Feedback 5. Completion

When you made your choices, were there any factors you did not consider? (please select all that apply)

- I did not consider clinical outcomes
- I did not consider patient experience
- I did not consider safety
- I did not consider spending per patient
- I did not consider distance
- I did not consider out of pocket costs
- I considered all six factors

Back Next

1. Introduction 2. About You 3. Choosing a Hospital 4. Survey Feedback 5. Completion

We are interested in understanding what was most important to you when choosing between hospitals.

Please rank from 1st (Most Important) to 6th (Least Important) the factors which affected your choices.

Response options

- Clinical Outcomes
- Patient Experience
- Safety
- Medicare Spending per Patient
- Distance
- Out of Pocket Cost

Your rankings

No responses have been ranked. Select them in the order that you would like to rank them from the "Response Options" list.

Clear rankings

Back Next

© 2023 Trenaman L et al. *JAMA Network Open.*

eTable 2. Geodemographic Benchmarks for Generating Weights for Medicare Population[a]

	Frequency	Percent
Race/Ethnicity by Gender		
Black Female	3,583,969	6.0%
Black Male	2,658,052	4.5%
Hispanic Female	3,010,462	5.1%
Hispanic Male	2,371,587	4.0%
White Female	23,937,827	40.2%
White Male	20,372,005	34.2%
Other[a] Female	2,050,164	3.5%
Other[a] Male	1,506,607	2.5%
Age Group by Race/Ethnicity		
Black 18-64	1,422,923	2.4%
Black 65-69	1,671,669	2.8%
Black 70-74	1,319,339	2.2%
Black 75+	1,828,090	3.1%
Hispanic 18-64	925,255	1.6%
Hispanic 65-69	1,433,898	2.4%
Hispanic 70-74	1,290,849	2.2%
Hispanic 75+	1,732,047	2.9%
White 18-64	4,614,839	7.8%
White 65-69	11,552,769	19.4%
White 70-74	10,783,196	18.1%
White 75+	17,359,028	29.2%
Other[b] 18-69	1,392,327	2.3%
Other[b] 70-74	882,268	1.5%
Other[b] 75+	1,282,176	2.2%
Race/Ethnicity by Education Attainment		
Black Less than high school diploma (or equivalent)	1,091,291	1.8%
Black High school diploma (or equivalent)	2,331,204	3.9%
Black Some College	1,635,206	2.8%
Black Bachelor or higher	1,184,320	2.0%
Hispanic Less than high school diploma (or equivalent)	1,952,083	3.3%
Hispanic High school diploma (or equivalent)	1,649,329	2.8%
Hispanic Some College	949,797	1.6%
Hispanic Bachelor or higher	830,840	1.4%
White Less than high school diploma (or equivalent)	3,240,183	5.5%
White High school diploma (or equivalent)	14,608,762	24.6%
White Some College	11,814,768	19.9%
White Bachelor or higher	14,646,120	24.6%
Other[b] Some College or below	2,204,947	3.7%
Other[b] Bachelor or higher	1,351,825	2.3%
Age Group by Gender		
18-64 Male	3,624,434	6.1%
18-64 Female	3,750,024	6.3%
65-69 Male	7,199,007	12.1%
65-69 Female	8,440,216	14.2%
70-74 Male	6,469,630	10.9%
70-74 Female	7,806,022	13.1%
75+ Male	9,615,180	16.2%

75+ Female	12,586,161	21.2%
**Race/Ethnicity**		
Asian American or Pacific Islander/Non-Hispanic	2,702,397	4.5%
Black/Non-Hispanic	6,242,021	10.5%
Hispanic	5,382,049	9.1%
White/Non-Hispanic	44,309,833	74.5%
Other[b] or 2+ Races/Non-Hispanic	854,374	1.4%
**Region**		
Northeast	10,848,760	18.2%
Midwest	12,754,083	21.4%
South	22,926,807	38.5%
West	12,961,025	21.8%
**Metropolitan Area**		
Non-Metro	10,342,465	17.4%
Metro	49,148,209	82.6%
**Household Income**		
Under $25,000	13,983,723	23.5%
$25,000-$49,999	15,045,948	25.3%
$50,000-$74,999	9,972,756	16.8%
$75,000-$99,999	6,333,289	10.7%
$100,000-$149,999	7,093,260	11.9%
$150,000 and over	7,061,699	11.9%
**Medicare coverage last year**		
Yes	59,490,675	100.0%
**Covered by private plan last year**		
Yes	26,034,788	43.8%
No	33,455,887	56.2%
**Public coverage last year**		
Yes	59,490,675	100.0%

[a] benchmarks are from the March 2021 Current Population Survey Annual Social and Economic Supplement (CPS – ASEC)
[b] 'Other' includes those that reported 'American Indian or Alaska Native' or 'Asian'

eTable 3. Unweighted and Weighted Respondent Characteristics

Characteristic	Unweighted %	Weighted %
Age group		
18-24	0%	0%
25-34	1%	1%
35-44	2%	2%
45-54	4%	3%
55-64	8%	7%
65-74	55%	50%
75+	31%	37%
Gender		
Female	51%	55%
Male	49%	45%
Race		
American Indian or Alaska Native	1%	1%
Asian	6%	4%
Black or African American	21%	11%
White	70%	83%
2+ races	2%	1%
Ethnicity		
Black, Non-Hispanic	20%	11%
Hispanic	20%	9%
Other[a], Non-Hispanic	6%	5%
White, Non-Hispanic	52%	75%
2+ Races, Non-Hispanic	1%	1%
Highest educational attainment		
No high school diploma or GED	7%	11%
High school graduate (high school diploma or the equivalent GED)	27%	32%
Some college or Associate's degree	32%	26%
Bachelor's degree	16%	15%
Master's degree or higher	18%	16%
Household income, $		
Less than $10,000	2%	4%
$10,000 to $24,999	14%	20%
$25,000 to $49,999	24%	25%
$50,000 to $74,999	19%	17%
$75,000 to $99,999	13%	11%
$100,000 to $149,999	14%	12%
$150,000 or more	13%	12%
Marital status		
Divorced	14%	14%
Never married	12%	11%

© 2023 Trenaman L et al. *JAMA Network Open.*

Now married	60%	57%
Separated	2%	2%
Widowed	12%	15%
Metro		
Metro	88%	83%
Non-Metro	12%	17%
Region		
Midwest	18%	21%
Northeast	16%	18%
South	43%	39%
West	23%	22%
Employment status		
Not working	85%	85%
Working full-time	5%	4%
Working part-time	10%	10%
Health insurance status		
Medicare, for people 65 and older, or people with certain disabilities	100%	100%
Medicaid, Medical Assistance, or another government-assistance plan	9%	10%
Insurance through a current or former employer or union	17%	16%
Insurance purchased directed from an insurance company	14%	16%
TRICARE or other military health care	5%	4%
VA (enrolled for VA health care)	5%	4%
Indian Health Service	0%	0%
Any other type of health insurance or health coverage plan	4%	5%

[a] 'Other' includes those that reported 'American Indian or Alaska Native' or 'Asian'

© 2023 Trenaman L et al. *JAMA Network Open.*

eTable 4. Results From Effects-Coded Mixed Logit Model

	β	SE	z	P>z	2.5%	97.5%
Clinical Outcomes						
☆	-1.89	0.12	-15.97	0.00	-2.12	-1.66
☆ ☆	-0.84	0.07	-12.11	0.00	-0.97	-0.70
☆ ☆ ☆	0.20	0.06	3.27	0.00	0.08	0.32
☆ ☆ ☆ ☆	1.17	0.08	15.43	0.00	1.02	1.31
☆ ☆ ☆ ☆ ☆	1.36	0.09	14.83	0.00	1.18	1.54
Patient Experience						
☆	-0.75	0.08	-9.40	0.00	-0.91	-0.59
☆ ☆	-0.40	0.07	-5.78	0.00	-0.53	-0.26
☆ ☆ ☆	0.10	0.06	1.53	0.13	-0.03	0.22
☆ ☆ ☆ ☆	0.40	0.08	5.11	0.00	0.25	0.55
☆ ☆ ☆ ☆ ☆	0.65	0.06	10.13	0.00	0.53	0.78
Safety						
☆	-0.88	0.09	-9.98	0.00	-1.05	-0.71
☆ ☆	-0.22	0.05	-4.18	0.00	-0.32	-0.12
☆ ☆ ☆	0.23	0.06	3.89	0.00	0.11	0.35
☆ ☆ ☆ ☆	0.29	0.06	4.49	0.00	0.16	0.41
☆ ☆ ☆ ☆ ☆	0.58	0.08	7.40	0.00	0.43	0.73
Efficiency						
☆	-0.24	0.06	-4.09	0.00	-0.36	-0.13
☆ ☆	-0.14	0.06	-2.39	0.02	-0.25	-0.03
☆ ☆ ☆	0.13	0.06	2.28	0.02	0.02	0.24
☆ ☆ ☆ ☆	0.01	0.07	0.16	0.88	-0.12	0.14
☆ ☆ ☆ ☆ ☆	0.24	0.06	3.90	0.00	0.12	0.36
Distance						
10 miles	-0.39	0.04	-9.34	0.00	-0.48	-0.31
100 miles	-0.01	0.04	-0.28	0.78	-0.09	0.07
200 miles	0.40	0.05	7.52	0.00	0.30	0.51
Out-of-Pocket Cost						
$200	-1.16	0.08	-14.96	0.00	-1.32	-1.01
$1,000	0.23	0.04	5.37	0.00	0.15	0.32
$4,000	0.93	0.06	15.41	0.00	0.81	1.05

Number of observations = 18,450
Log likelihood = -4215.8285
AIC = 8511.657

eTable 5. Results From Effects-Coded Mixed Logit Model (‘Consistent’ Subsample)

	β	SE	z	P>z	2.5%	97.5%
Clinical Outcomes						
☆	-2.02	0.15	-13.87	0.00	-2.30	-1.73
☆ ☆	-0.95	0.08	-12.00	0.00	-1.10	-0.79
☆ ☆ ☆	0.21	0.07	3.00	0.00	0.07	0.35
☆ ☆ ☆ ☆	1.27	0.09	14.33	0.00	1.10	1.44
☆ ☆ ☆ ☆ ☆	1.48	0.12	12.67	0.00	1.25	1.71
Patient Experience						
☆	-0.80	0.10	-7.83	0.00	-1.00	-0.60
☆ ☆	-0.40	0.07	-5.40	0.00	-0.55	-0.26
☆ ☆ ☆	0.06	0.07	0.83	0.41	-0.08	0.21
☆ ☆ ☆ ☆	0.41	0.10	4.30	0.00	0.22	0.60
☆ ☆ ☆ ☆ ☆	0.74	0.07	9.96	0.00	0.59	0.88
Safety						
☆	-1.01	0.10	-10.51	0.00	-1.20	-0.83
☆ ☆	-0.25	0.06	-3.89	0.00	-0.37	-0.12
☆ ☆ ☆	0.31	0.07	4.19	0.00	0.16	0.45
☆ ☆ ☆ ☆	0.31	0.08	4.12	0.00	0.16	0.46
☆ ☆ ☆ ☆ ☆	0.64	0.09	6.74	0.00	0.45	0.82
Efficiency						
☆	-0.28	0.07	-4.07	0.00	-0.42	-0.15
☆ ☆	-0.23	0.07	-3.45	0.00	-0.36	-0.10
☆ ☆ ☆	0.18	0.07	2.69	0.01	0.05	0.30
☆ ☆ ☆ ☆	0.01	0.08	0.16	0.87	-0.14	0.16
☆ ☆ ☆ ☆ ☆	0.32	0.07	4.69	0.00	0.19	0.46
Distance						
10 miles	-0.41	0.05	-7.87	0.00	-0.52	-0.31
100 miles	0.00	0.05	0.05	0.96	-0.09	0.09
200 miles	0.41	0.06	6.63	0.00	0.29	0.53
Out-of-Pocket Cost						
$200	-1.21	0.08	-14.51	0.00	-0.14	-1.04
$1,000	0.22	0.05	4.26	0.00	0.12	0.33
$4,000	0.98	0.07	14.51	0.00	0.85	1.12

Number of observations = 15,750
Log likelihood = -3542.8902
AIC = 7165.78

© 2023 Trenaman L et al. *JAMA Network Open.*

eTable 6. Results From Effects-Coded Mixed Logit Model (‘Understood’ Subsample)

	β	SE	z	P>z	2.5%	97.5%
Clinical Outcomes						
☆	-2.05	0.13	-15.45	0.00	-2.31	-1.79
☆ ☆	-0.95	0.08	-11.44	0.00	-1.11	-0.79
☆ ☆ ☆	0.25	0.08	3.30	0.00	0.10	0.39
☆ ☆ ☆ ☆	1.24	0.09	13.23	0.00	1.06	1.42
☆ ☆ ☆ ☆ ☆	1.51	0.12	12.67	0.00	1.27	1.74
Patient Experience						
☆	-0.76	0.09	-8.53	0.00	-0.94	-0.59
☆ ☆	-0.37	0.07	-4.95	0.00	-0.51	-0.22
☆ ☆ ☆	0.11	0.07	1.67	0.10	-0.02	0.24
☆ ☆ ☆ ☆	0.38	0.09	4.40	0.00	0.21	0.54
☆ ☆ ☆ ☆ ☆	0.64	0.07	9.27	0.00	0.51	0.78
Safety						
☆	-0.93	0.09	-10.29	0.00	-1.11	-0.75
☆ ☆	-0.22	0.06	-3.89	0.00	-0.34	-0.11
☆ ☆ ☆	0.23	0.07	3.57	0.00	0.10	0.36
☆ ☆ ☆ ☆	0.35	0.08	4.53	0.00	0.20	0.50
☆ ☆ ☆ ☆ ☆	0.57	0.08	7.14	0.00	0.42	0.73
Efficiency						
☆	-0.25	0.07	-3.79	0.00	-0.38	-0.12
☆ ☆	-0.15	0.06	-2.23	0.03	-0.27	-0.02
☆ ☆ ☆	0.14	0.07	2.04	0.04	0.01	0.27
☆ ☆ ☆ ☆	0.00	0.08	-0.05	0.96	-0.15	0.15
☆ ☆ ☆ ☆ ☆	0.26	0.07	3.73	0.00	0.12	0.40
Distance						
10 miles	-0.36	0.46	-7.75	0.00	-0.45	-0.27
100 miles	0.01	0.04	0.24	0.81	-0.07	0.09
200 miles	0.35	0.06	6.31	0.00	0.24	0.46
Out-of-Pocket Cost						
$200	-1.23	0.09	-13.94	0.00	-1.40	-1.06
$1,000	0.26	0.05	5.38	0.00	0.16	0.35
$4,000	0.97	0.07	14.18	0.00	0.84	1.11

Number of observations = 16,216
Log likelihood = -3542.3579
AIC = 7164.716

© 2023 Trenaman L et al. *JAMA Network Open.*
