# Supplementary material for: Medicare Beneficiaries’ Perspectives on the Quality of Hospital Care and Their Implications for Value-Based Payment
Source: JAMA Netw Open. 2023 Jun 21;6(6):e2319047. doi: 10.1001/jamanetworkopen.2023.19047 (PMC10285577; doi:10.1001/jamanetworkopen.2023.19047)
Supplement: Supplement 2. — Data Sharing Statement [file jamanetwopen-e2319047-s002.pdf]

Data Sharing Statement

Trenaman. Medicare Beneficiaries' Perspectives on the Quality of Hospital Care and Their Implications for Value-Based Payment. *JAMA Netw Open*. Published June 21, 2023. doi:10.1001/jamanetworkopen.2023.19047

Data
Data available: Yes
Data types: Deidentified participant data, Data dictionary
How to access data: Data are available by contacting the lead author at ltrenaman@cheos.ubc.ca
When available: With publication

Supporting Documents
Document types: None

Additional Information
Who can access the data: Researchers whose proposed use of the data has been approved.
Types of analyses: For academic purposes.
Mechanisms of data availability: With signed data access agreement.
Any additional restrictions: None.
